# Intravascular Papillary Endothelial Hyperplasia of the Orbit: A Case of Masson’s Tumor

**DOI:** 10.7759/cureus.6266

**Published:** 2019-12-01

**Authors:** Stephen C Dryden, Shane Marsili, Andrew G Meador, M. Barry Randall, Brian Fowler

**Affiliations:** 1 Ophthalmology, University of Tennessee Health Science Center, Memphis, USA; 2 Ophthalmology, University of Nevada, Reno School of Medicine, Reno, USA; 3 Dermatology and Pathology, University of Tennessee Health Science Center, Memphis, USA

**Keywords:** masson's tumor, ipeh, intravascular papillary endothelial hyperplasia, ophthalmology, oculoplastics, oncology, oncological surgery

## Abstract

Herein, we present a case of a 64-year-old male presenting with diplopia that was found to have a left orbital Masson’s tumor. It is necessary to differentiate it from malignant angiosarcoma as complete excision is often curative. The following case emphasizes the modalities that can be used for diagnosis as well as the appropriate treatments.

## Introduction

Masson’s tumor, also known as intravascular papillary endothelial hyperplasia (IPEH), is a rare benign neoplasm that affects the head, neck, and upper extremities. There are few (less than 20) periorbital cases that have been reported in ophthalmologic literature [1-4]. Masson’s tumor can often be difficult to distinguish between other entities including malignant angiosarcoma; its prompt diagnosis is important to begin optimal treatment as soon as possible.

## Case presentation

A 63-year-old Caucasian male with past medical history of diabetes mellitus and past ocular history of bilateral cataract extraction with intraocular lens (CEIOL) presented to an outside vitreoretinal specialist one-month post-CEIOL due to diplopia and possible retinal detachment. Best corrected visual acuity (BCVA) was measured as 20/25 OU, pupils were reactive without afferent pupillary defect (APD) OU, and intraocular pressure (IOP) was measured as 21 and 18 mmHg, respectively. Optical coherence tomography of both eyes revealed a flat and attached macula OU. Fundus photos revealed irregular appearance of nasal retina OS. The patient was subsequently diagnosed with nasal choroidal effusion and was given cyclopentolate, prednisolone, and oral dexamethasone.

The patient returned for follow-up one week later with no resolution of symptoms and new complaint of an inferior “knot” OS with pain and pressure. BCVA and pupillary examination were unchanged. IOP was measured as 16 and 18 mmHg, respectively. Dilated fundoscopic examination was performed, and treatment was discontinued as the choroidal effusion had resolved.

**Figure 1:**
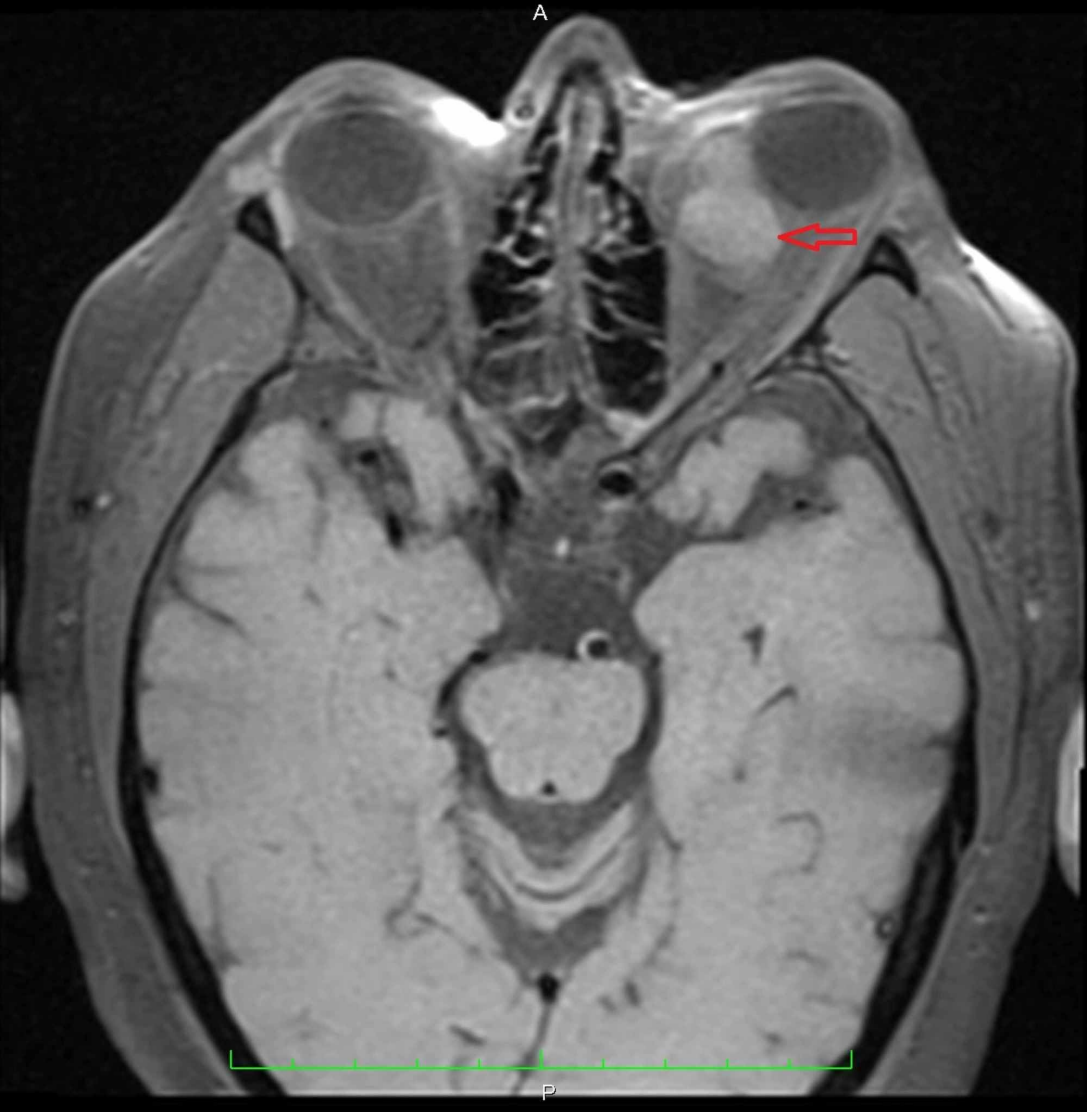
Transverse MRI of the orbits without contrast revealed a mass in the left orbit contributing to proptosis

**Figure 2:**
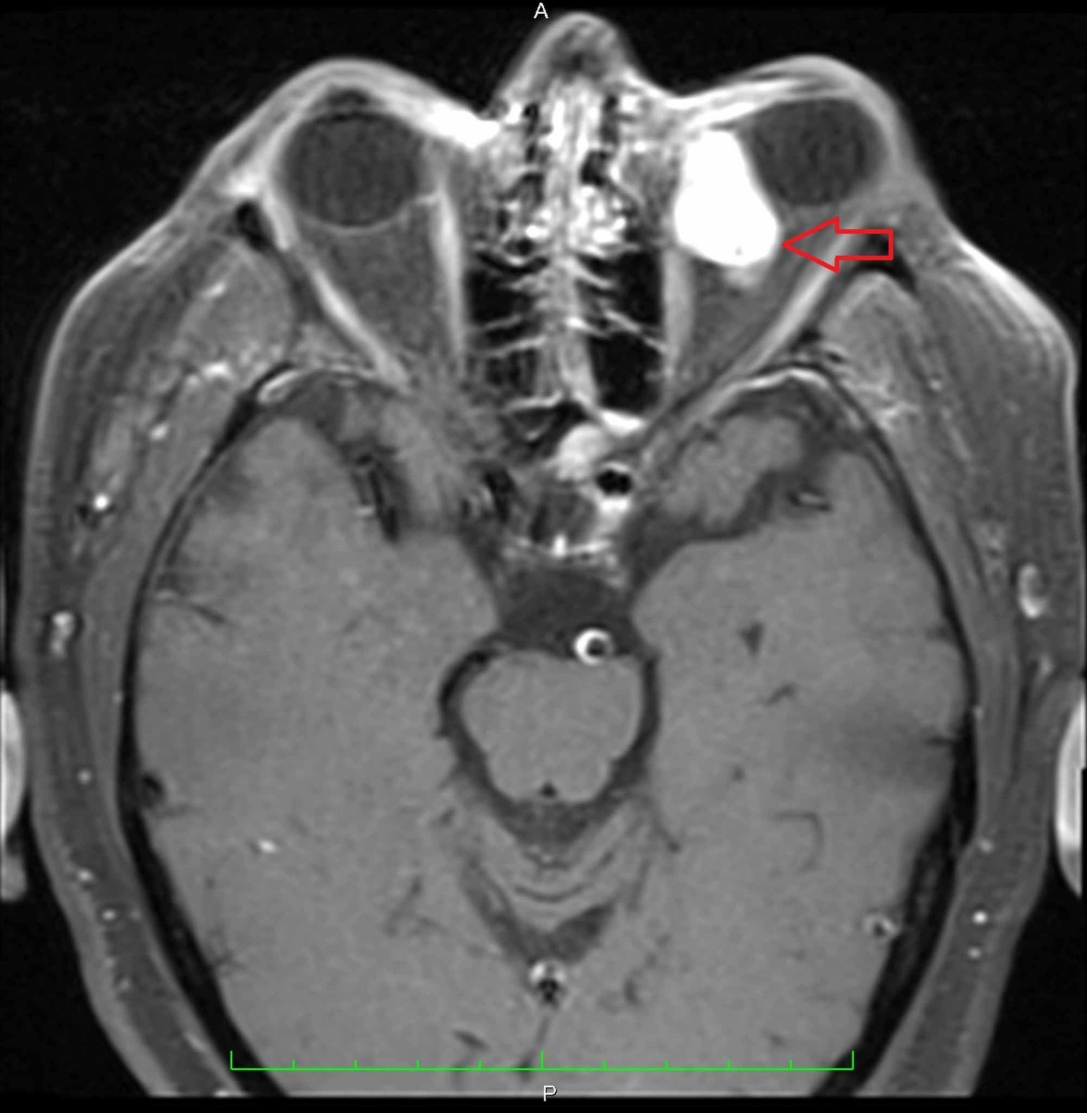
Transverse MRI of the orbits with contrast revealed an enhancing lesion in the left orbit involving both the intraconal and extraconal compartments measuring 2.5 x 1.6 x 3.2 cm

**Figure 3:**
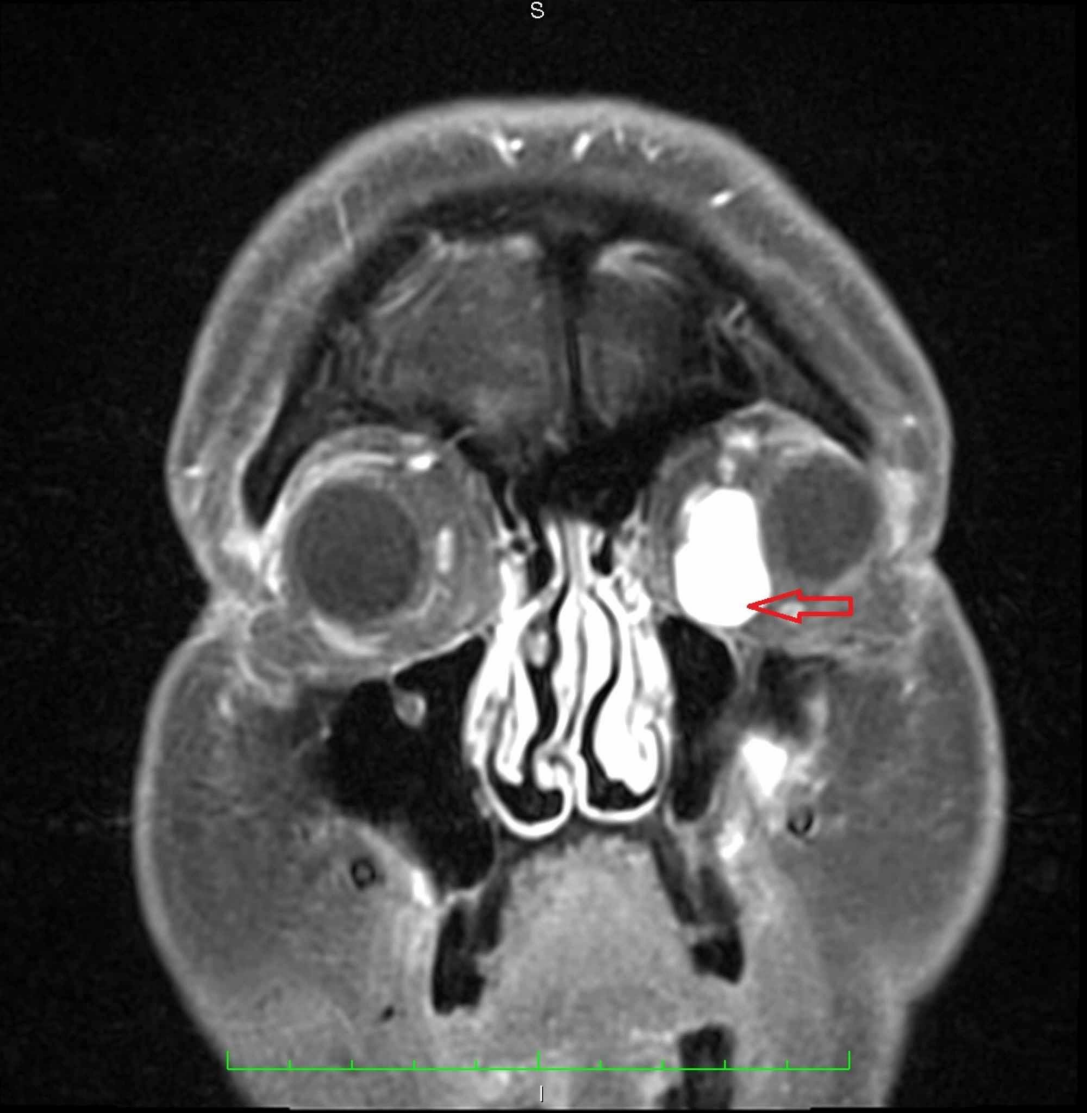
Coronal MRI of the orbits with contrast gives another view of the enhancing lesion within the left orbit

Further follow-up three weeks later revealed no change in subjective pain and pressure symptoms OS. The patient described his diplopia as horizontal and binocular. Further questioning revealed that his symptoms began two months ago after CEIOL. BCVA, pupillary examination, and IOP were unchanged. Dilated fundoscopic examination was notable for a return of his choroidal effusion. MRI of the orbits with and without contrast ordered which revealed an enhancing 2.5 x 1.6 x 3.2 cm left orbital lesion involving both the intraconal and extraconal compartments and contributing to the left-sided proptosis (Figures 1-3).

**Figure 4:**
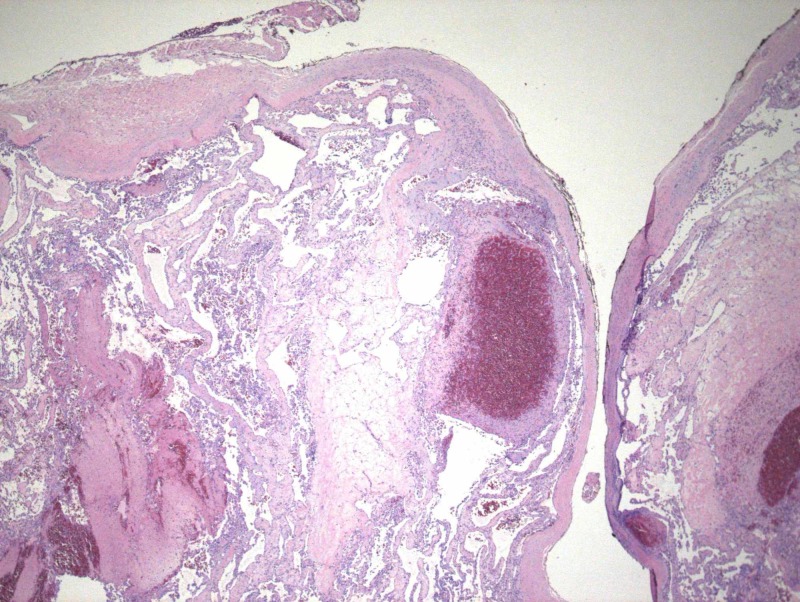
The absence of tissue necrosis, noninfiltrative intraluminal location of the lesion, and the association between proliferating tuft structures with thrombotic material differentiate IPEH from angiosarcoma

**Figure 5:**
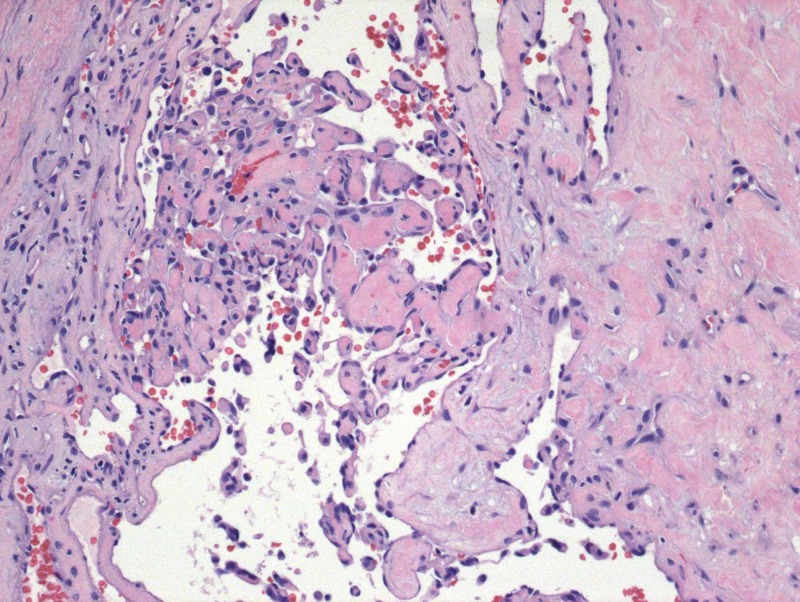
Papillary formations and arborizing channels

**Figure 6:**
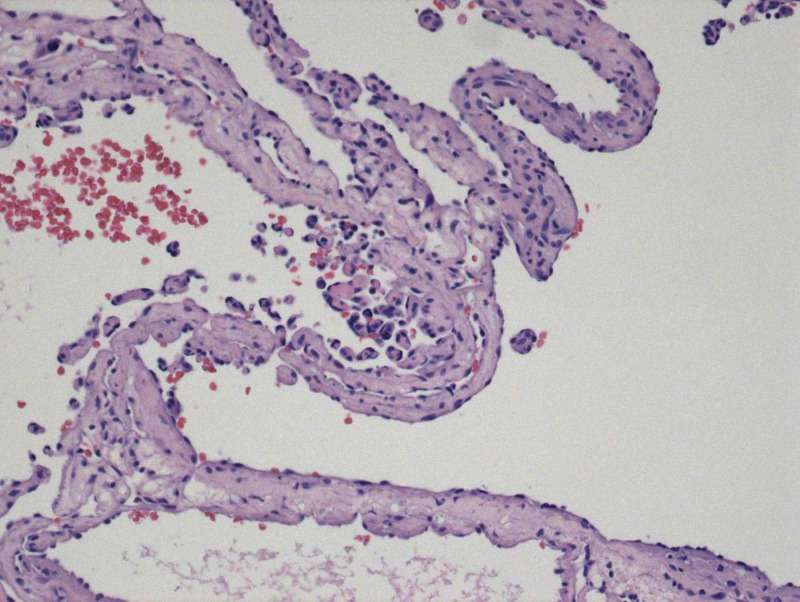
Vascular channels containing erythrocytes and serum

**Figure 7:**
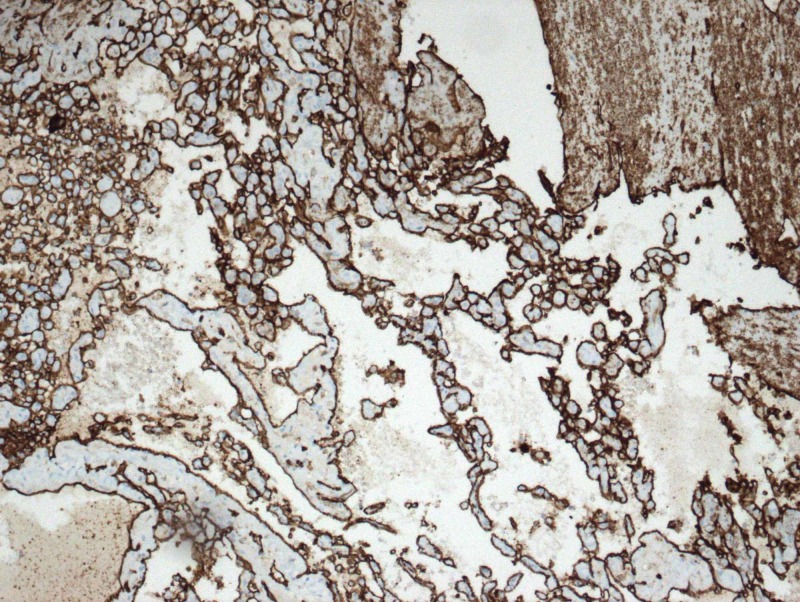
CD31 immunohistochemical marker

At this time, the patient was referred to oculoplastics for further evaluation. Nineteen days after the MRI, the patient reported continued diplopia, pain, and proptosis OS. His ocular examination was as follows: BCVA of 20/25 OU, pupils reactive with no APD OU, IOP of 21 and 27 mmHg, respectively. His extraocular movements were full OD and -1 in all gazes OS. His MRI was reviewed, and the patient was scheduled for a biopsy of the orbital mass. An approximately 2.1 x 0.5 cm mass submitted for routine sectioning revealed papillary formations and arborizing channels contained within a fibromuscular wall compatible with a dilated vascular space. Variably sized vascular channels intersect the interior of the mass, and these channels contain erythrocytes and serum. Papillae are characterized by a hyalinized fibrovascular core lined by plump mononuclear cells that occasionally form syncytia. CD31 immunohistochemical marker for endothelial cells richly outlines the furcating vascular network (Figures 4-7).

The final diagnosis was IPEH, also known as Masson’s tumor.

The patient subsequently underwent orbitotomy with excision with removal of approximately 80% of the tumor. The other 20% was inaccessible as it was circumferential to the optic nerve. The patient had improvement of pain, diplopia, and proptosis post-surgery, and a follow-up CT was scheduled three months later.

## Discussion

IPEH was first described by Masson in 1923 as “vegetate intravascular hemangioendothelioma”[5]. It is characterized by a reactive hyperplastic proliferation of the vascular endothelium [1]. It has been speculated that the primary process is endothelial proliferation followed by thrombus formation; however, others conjecture that endothelial papillary proliferation is a response to thrombus formation [1-3]. There is no age, race, or sex predilection for the generalized IPEH; however, in their case series, Wagh et al found that the average age for periorbital IPEH was 41.2 years with a male to female ratio of 2:1 [1,2].

IPEH has a predisposition for the head, neck, and upper extremities, and it may present in both the orbit and eyelid. It often presents as a firm, cystic, freely mobile, reddish/purple swelling that may increase in size [3,4]. The differential diagnosis of IPEH includes vascular lesions such as angiosarcoma, angiolymphatic hyperplasia with eosinophilia (Kimura disease), and other primary or secondary tumors [1,2,6]. The histopathologic absence of tissue necrosis, noninfiltrative intraluminal location of the lesion, and the association between proliferating tuft structures with thrombotic material help to differentiate IPEH from angiosarcoma [1,7]. It needs to be differentiated from malignant angiosarcoma as complete excision is often curative [8].

There are three forms of IPEH. Primary IPEH forms within a dilated vascular space with a preference for extremities. Secondary or reactive IPEH is the most common and is predominately found in the musculature. It originates within pre-existing vascular malformations such as varix, lymphangioma, or cavernous hemangioma. Tertiary IPEH is the rarest, forms within hematomas, and is extravascular.

Evidence of unilateral proptosis in an elderly patient should prompt imaging to rule out mass effect. MRI is the preferred imaging technique for identifying IPEH. Lesions appear isointense on T1-weighted imaging and hyperintense on T2-weighted imaging with homogenous enhancement following contrast administration [1]. Management depends on the clinical features and progression of the lesion. Surgical excision is the preferred method of treatment; however, the lesion may recur with incomplete excision. There may be a role for adjuvant chemotherapy and radiotherapy for recurrent or concerning lesions [1-3].

## Conclusions

Recognizing IPEH and differentiating from malignant lesions such as angiosarcoma is important to prevent morbidity related to unnecessary chemotherapy, radiation, and surgery. This case emphasizes the importance of early diagnosis and a strong referral base for appropriate diagnosis and treatment. Even in cases where surgical correction is not entirely curative, a significant improvement of symptoms can be obtained post-surgery. This patient received appropriate care and surgical correction to remove 80% of the tumor, and he subsequently had significant quality-of-life improvements. 
